# P-99. Defining Success and Failure in Prosthetic Joint Infections: A Meta-epidemiologic Study Towards a Core Outcome Set

**DOI:** 10.1093/ofid/ofaf695.328

**Published:** 2026-01-11

**Authors:** Seyed Mohammad Amin Alavi, Francesco Petri, Fabio Borgonovo, Haseeb Khawar, Matteo Passerini, Takahiro Matsuo, Anil Jagtiani, Andrea Gori, Ben Marson, Elie F Berbari

**Affiliations:** Ahvaz Jundishapur University of Medical Sciences, Ahvaz, Khuzestan, Iran; Mayo Clinic, Rochester, Minnesota, Rochester, Minnesota; Mayo Clinic, Rochester, Minnesota; University Hospitals Nottingham NHS Trust, Nottingham, UK, Nottingham, England, United Kingdom; University of Milan, Milan, Italy, Milan, Lombardia, Italy; Mayo Clinic, Rochester, Minnesota; Kaiser Permanente Southern California, Fontana, CA, USA., Fontana, California; Infectious Diseases and Immunopathology, Department of Clinical Sciences, Università di Milano, Luigi Sacco Hospital, Milan, Italy, Milano, Lombardia, Italy; University Hospitals Nottingham NHS Trust, Nottingham, UK, Nottingham, England, United Kingdom; Mayo Clinic, Rochester, Minnesota

## Abstract

**Background:**

A unified definition of prosthetic joint infection outcomes (PJI) is crucial for consistent data, study comparisons, and better care. This study systematically reviews how PJIs outcomes are defined, building on our prior research on native vertebral osteomyelitis and postoperative spinal infection.

**Figure 1:**
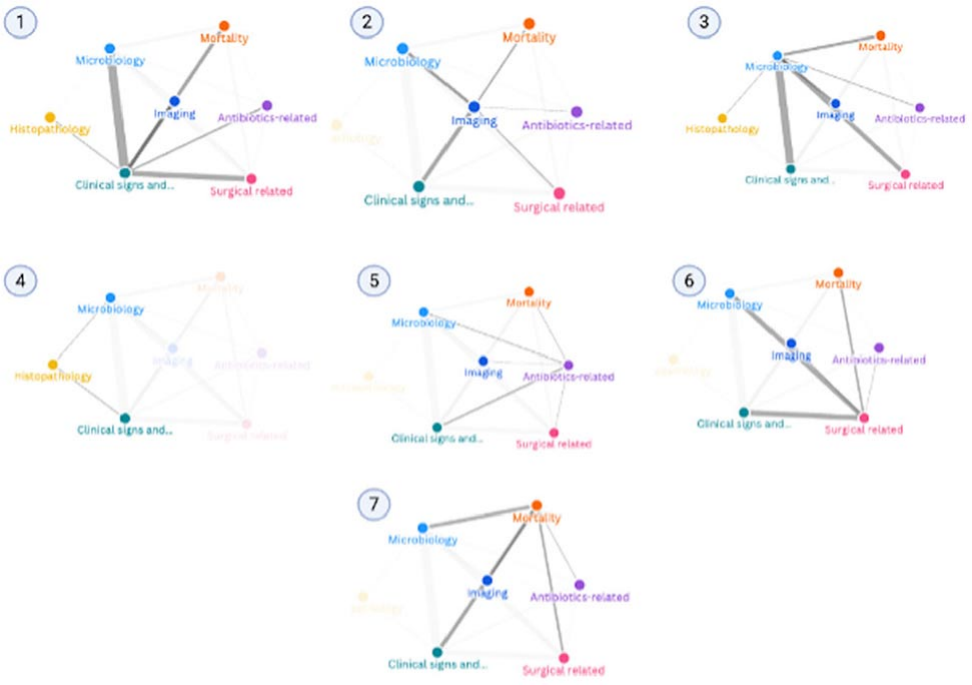
Network plot illustrating the co-occurrence of outcome criteria in prosthetic joint infection studies. Edge thickness reflects the frequency of the co-occurrence of each criterion. Each plot highlights one criterion, namely (1) clinical signs, (2) imaging, (3) microbiology, (4) histopathology, (5) antibiotics-related, (6) surgical-related, (7) mortality.

**Methods:**

We conducted a comprehensive search of the Cochrane, Embase, Medline, and Scopus databases for studies published after 2005 that clearly defined outcomes for PJI. Eligible studies included randomized controlled trials and cohort studies with a minimum of 10 patients diagnosed with PJI. Our methodology adhered closely to the PRISMA guidelines. Using a saturation approach, we extracted and grouped explicit definitions of PJI outcomes into thematic criteria. A network plot (Figure 1) was used to visualize how frequently these criteria appeared and how they co-occurred across the included studies.

**Results:**

Out of 7,584 screened studies, 552 (7.3%) met the inclusion criteria. This pilot analysis included 49 randomly selected studies (8,162 patients), mostly retrospective (48/49, 98%), focusing on mixed joint replacements (25/49, 51%), two-stage exchanges (15/49, 31%), or mixed surgeries (24/49, 49%). Only 6/49 (12.2%) applied a society-endorsed definition, and 13/49 (26.5%) cited a reference for their outcome definitions. Overall, 32/49 (65.3%) explicitly defined "success," 35/49 (71.4%) defined "failure," and 16/49 (32.7%) defined both. Most studies (25/49, 51%) had follow-up periods over two years, and 14/49 (28.6%) included patient-reported outcomes, identifying 14 unique PROMs (most commonly UCLA, HHS, and KSS scores). Functional outcomes were assessed in 13/49 (26.5%) studies. We identified 16 distinct outcome criteria across seven categories: clinical signs, microbiology, imaging, histopathology, antibiotics-related, surgical-related, and mortality. Network analysis revealed strong links among clinical signs, microbiology, surgical factors, mortality, and imaging.

**Conclusion:**

Our pilot study found major variation in PJI outcome definitions, with few standardized criteria. A consensus-driven approach is needed to strengthen evidence synthesis and advance patient care in PJI.

**Disclosures:**

All Authors: No reported disclosures

